# The immunological subtypes of cone photoreceptors in mouse and primate retinas

**DOI:** 10.1371/journal.pone.0362261

**Published:** 2026-11-13

**Authors:** Vivian Y. Pang, Zhuo Yang, Samuel M. Wu, Ji-Jie Pang

**Affiliations:** Department of Ophthalmology, Baylor College of Medicine, Houston, Texas, United States of America; University of California Berkeley, UNITED STATES OF AMERICA

## Abstract

We studied cone photoreceptors in adult mouse and baboon retinas for their structure and diversity with immunomarkers and confocal microscopy. In mouse retinas, L/M-opsin and S-opsin antibodies labeled regular cones primarily in the outer segment (OS). In dark-adapted conditions, S-opsin-positive OSs (152,941/retina) showed a Gaussian distribution with a density range of 40,245–150/mm^2^ ventrodorsally and 19,072–184/mm^2^ temporonasally. ~ 10% somas of L/M-opsin-positive cones were attached as pairs. 7.6% of L/M-opsin-expressing cones and 5.7% of S-opsin-positive cones showed higher opsin levels in soma and axon, and we named them *lm*-cones or *ss*-cones, respectively, which possessed single or dual OS(s), indicating the presence of double-cones and more color-sensitive subtypes. Unlike regular cones, *lm*-cones had significantly smaller OSs and shorter inner segments (ISs), and *ss*-cone somas resided in rod soma rows with a much longer IS-soma distance. Retrogradely labeling of retinal ganglion cells (RGCs) revealed two photoreceptor-RGC hybrids with OSs. Peanut agglutin (PNA) identified two OS calibers: 1) 1–2 μm with two intertwined continuous PNA-positive strings and 2) 2–3μm with 3–4 PNA-positive strings, which preferred the central or peripheral retina and matched one or two OS compartments, respectively. PNA-positive strings were thinner in ISs. In monkey cones, the OS disk region was positive for PNA (89.5%) or calcium-binding protein 5 (10.5%). OS surface was covered by vertical PNA-positive grids, which were thinner in ISs, absent in fovea cone OSs, and multi-barrel-like in other cones. PNA-positive sheath contained up to ~10–12 parallel grids of similar thickness, distance, and immunoreactivity, likely serving as the wall of cones.

## Introduction

Retinal cone photoreceptors have several subtypes of different photopigments and spectral sensitivity, and the cone subtype, location, and density are fundamental factors determining day vision and color vision. The subtypes and distribution of mouse cones show some similarities to primate cones. At eccentricities that correspond to the peripheral half of the human macula, the rod-to-cone ratio is similar between human and mouse over most of this region [1], though the actual photoreceptor cell density is much greater in the mouse central retina than in the human macula. In the mouse retina, the cone population has been shown to have a general distribution peak in the central retina [2,3], and the average cone density was reported to be 13000/mm^2^ at 82 degrees and 6000/mm^2^ at 0 degrees. The paracentral cone density was reported to be ~ 1/2 of the peak density, and the peripheral cone density was 1/4 of the peak density with a total population of ~160,000–180,000 per retina and the maximum density of 16500/mm^2^ [2].

Retinal cone photoreceptors are primarily classified by their photopigment. Primate retinas possess three subtypes of cones, which express the long-wavelength opsin (L-cones), medium-wavelength opsin (M-cones), or short-wavelength opsin (S-cones). On the other hand, calcium-binding proteins are critical for calcium signaling in neurons and widely used for identifying subtypes of retinal bipolar (BCs), horizontal (HCs), amacrine (ACs), and ganglion cells (RGCs) [4–7]. Calbindin labels cones in most primates except for outer segments (OSs) [5,8], serving as a classic marker for cones. Peanut agglutinin (PNA) is another biomarker widely used to identify cones. It labels the cone matrix sheath as well as OSs [9–11]. Calbindin- and PNA-labeled cones are presumably identical, but some cone OSs can be either positive for calbindin or calretinin [5,7], suggesting more functional subtypes of cones in addition to the subtypes purely determined by photopigments. The uniformity of immunoreactivity for PNA, calbindin, and the calcium-binding protein 5 (CaBP5) among baboon cones has been unclear.

In the mouse retina, most individual cones express both M- and S-opsin [12,13]. Some cones may dominantly express either M- or S-opsin, known as M-cones or S-cones, accordingly. 3–10% mouse cones express just S-opsin [14,15], and S-opsin shows a dorsal-ventral gradient [12,13]. The dorsal-ventral and temporal-nasal gradients of S-opsin have not been fully quantified in the mouse retina. Better defining the subtypes of cones and their distribution is a fundamental task for visual neuroscience. First, subtypes and distribution of cones can determine the function and distribution of bipolar cells. Researchers have accepted a uniform distribution of different subtypes of retinal neurons as a basic rule for location-independent visual perception [16,17]. However, the uneven distribution of S-opsin appears to cause the uneven distribution of the postsynaptic bipolar cells [15] to violate this rule. Second, mouse cones are small, and the morphological or immunological subtypes of M-, S-, or M/S-cones beyond the subtypes classified by photopigments have not been observed. Besides, the double cone is a common cone subtype of unique functions in vertebrates like fishes, reptiles, and birds [18–20], which has two OSs. Double cones have not been reported in the mouse or monkey retina [21,22].

Cones are long cells, and the OS of mammalian cones is often presented by a comb-like stack of discs connected to the IS by a thin connecting cilium. This structure suggests their vulnerability to physical stress. Cone degeneration occurs in retinal diseases, such as macular degeneration, and can cause blindness. Its pathological mechanism has been unclear. Cones, different from most retinal neurons, have a PNA-positive sheath covering most cone OS, and the distal portion of OSs may contact processes of the retinal pigment epithelium for regeneration [22–24]. Later studies have indicated the involvement of the PNA-positive sheath [25–27] and retinal pigment epithelium in cone degenerative diseases. PNA combines glycoproteins ranging from 30 to 88 kDa in various species [28], and the PNA-labeled “cylindrical domain” of cones has been defined as the “extracellular matrix sheath” as a portion of the retinal interphotoreceptor matrix (IPM) since 1986 [27–30]. IPM is less electron-dense. Under an electron microscope, IPM was reported to contain fine granular and filamentous materials [31], but EM did not reveal any specific structure for the PNA-positive cone sheath [11]. Except for the IPM, PNA also specifically labels OS disks of cones and synaptic regions of cone pedicles and telodendrites [9–11,16,32]. Such immunological similarity between cones and their PNA-positive sheath has not been further evaluated, and the structural variation of the PNA-positive sheath among cones has not been reported.

In this work, we investigated cones from mouse and monkey retinas with immunomarkers and confocal microscopy. We quantified the distribution gradient of mouse S-opsin-positive cones, identified several novel cone subtypes in the mouse and monkey retina, and revealed unique and variable structures for the PNA-positive sheath among cones in the mouse and primate retina.

## Results

### Four novel morphological subtypes of cones were identified from the mouse retina

In mouse retinal slices (n, 24 animals) triple-labeled for PNA, L/M-opsin, and TO-PRO-3 (Fig 1), cone OSs positive to L/M-opsin were also immunoreactive to PNA (98%). L/M-opsin immunoreactivity (IR) was concentrated in the middle portion of OSs in all cones, resembling M-cones previously identified [14,15]. However, in a minority of cones, L/M-IR clearly revealed the IS, somatic membrane, and the axon (Table 1). We specifically referred to the former as regular cones and the latter as *lm*-cones, which took 92.4% and 7.6% (6/79), respectively, of all cones positive to PNA. These *lm*-cones showed normal anatomical features at the soma, axon, and nucleus.

**Table 1:** Immunology of two novel subtypes of cones in the mouse retina.

			OS	IS	Soma	Axon stem	Axon Terminal	OS counts	Soma Pair
L/M-opsin-positive cone									10%
Regular cone		PNA	+++	+	+	-/+	+++	1	
		L/M-opsin	+++	+	+	+	++		
*lm*-cone		PNA	+++	+	+	-/+	+++	1-2	
		L/M-opsin	++	++	++	++	+++		
S-opsin-positive cone								*n.a.
Regular cone		PNA	+++	+	+	-/+	+++	1	
		S-Opsin	+++	–	–	–	–		
*ss-cone*		PNA	+++	+	+	-/+	+++	1-2	
		S-opsin	++	++	++	++	+++		
Previously identified cone types									
M/S-cone		PNA	+++					1	0
		L/M-opsin	+ to +++						
		S-opsin	+ to +++						
M-cone		PNA	+++					1	0
		L/M-opsin	+++						
		S-opsin	–						
S-cone		PNA	+++					1	0
		L/M-opsin	–						
		S-opsin	+++						

*n.a.: S-opsin signals did not reveal the somas of most cones.

**Fig 1:**
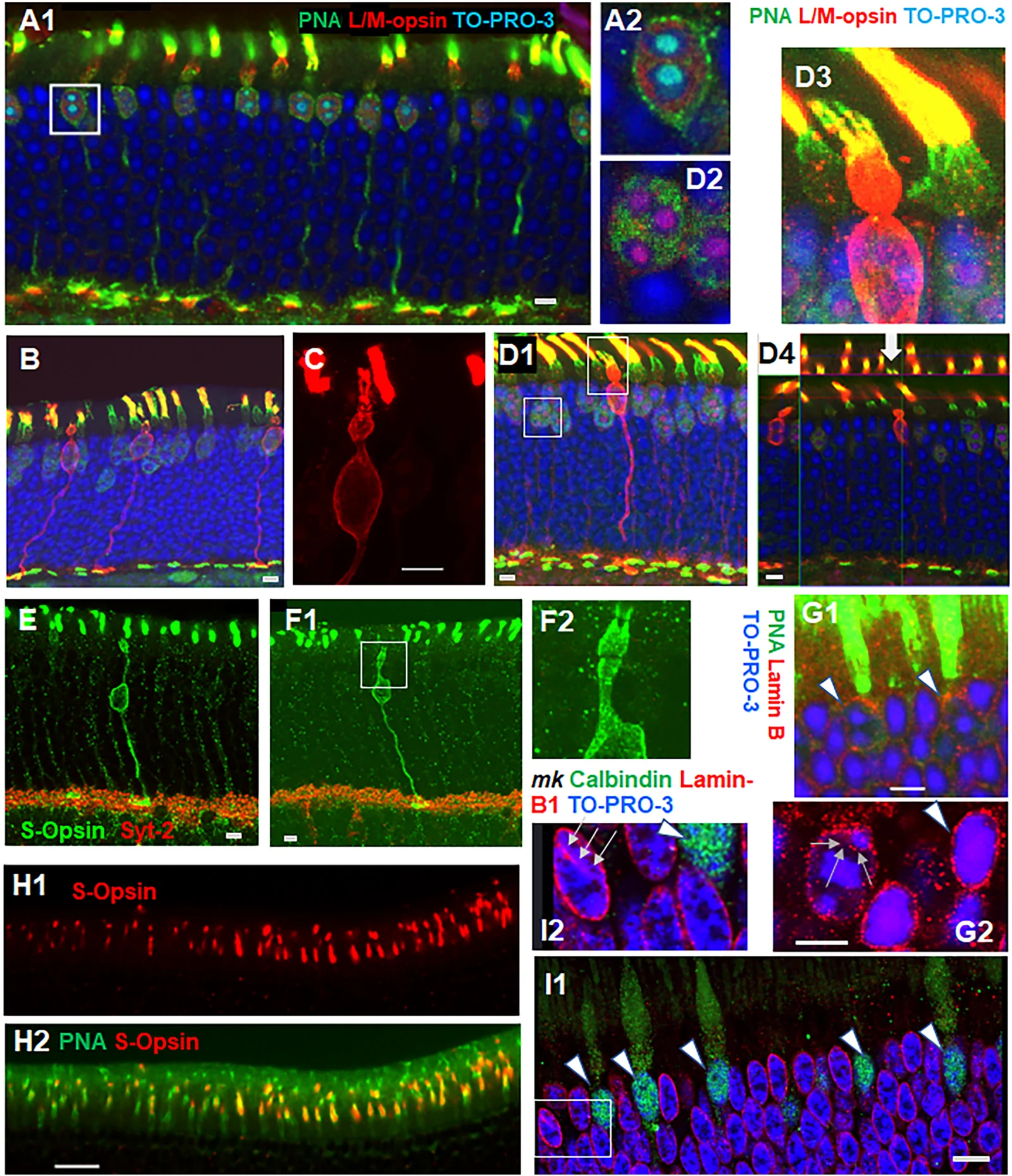
Morphological and immunological subtypes of cones in the mouse retina. **A-G and I**: Stacked confocal images each from a series of optical sections. **A-D**: Mouse retinal slices triple-labeled for PNA (green), L/M-opsin (red), and TO-PRO-3 (blue). PNA intensively labels the outer segment (OS) and axonal terminals of regular cones and weakly labels their inner segment (IS), soma, and axon stem. L/M-opsin-positive OSs are also PNA-labeled. In the OS, PNA signals wrap the L/M-opsin signal. In IS, soma, and axon, PNA signals are primarily inside the L/M-positive plasma membrane. Each cone soma may contain 1-4 nuclear regions positive for TO-PRO-3, and each nucleus contains a large nucleolus positive for both TO-PRO-3 and L/M-opsin (A2 and D2). **B-D:** Some cones were entirely labeled for L/M-opsin, namely, *lm*-cones (red). The *lm*-cones show a much shorter and rounder IS and one (B) or two OS(s) (C, D1, 3, and 4, arrow), and their OSs and nucleoli are less immunoreactive for L/M-opsin compared with regular cones. **D4**: Orthographic projection of confocal images showing the two OSs (arrow) of the *lm*-cone in D1 and D3. **E-F**: Two representative *ss*-cones. S-opsin antibody reveals OSs of mouse cones. The *ss*-cones are fully labeled, showing a narrow IS, a long IS-soma distance (i.e., myoid), and one (E) or two OS(s) (F2). **G**: The mouse retina was labeled for PNA, Lamin-B1, and TO-PRO-3. The immunoreactivity (IR) of Lamin-B1 is punctate (red), which envelopes cone nuclei (white arrowhead) without dividing nuclear regions (G1) except for some cones (G2, triple arrow). **H**: A long retinal slice labeled for PNA (green) and S-opsin (red) under low magnification. **I**: Lamin-B1-IR does not reveal nuclei of the monkey (*mk*) cones identified by calbindin D-28K (I1, white arrowhead). It identifies the nuclear membrane of other photoreceptors and occasionally separates nuclear regions for putative cone somas in the first row of the outer nuclear layer (I2, triple arrow). Scale bar is 20 μm in H and I, and 5 μm for the rest.

Somas of *lm*-cones and regular cones resided in the first soma row in the outer nuclear layer (ONL), and those of *lm*-cones were the closest to the outer limiting membrane. The IS of *lm*-cones was significantly rounder and shorter, which was situated right above the soma with a much shorter IS-soma distance (Fig 1 and Tables 1 and 2), and their OSs and nucleoli were less immunoreactive for L/M-opsin, compared with regular cones. Furthermore, we observed that *lm*-cones possessed one or two shorter OSs weakly labeled for L/M-opsin (Figs 1C, 1D and 2E), indicating novel single and double *lm*-cone subtypes. M-cones and S-cones have been previously distinguished by the photopigment in OSs instead of the cell morphology, and they have not been known to contain double cones.

**Table 2:** The morphology of two novel subtypes of cones in the mouse retina.

		Regular cone	*lm*-cone	*ss*-cone
OS	length, μm	11.26 ± 0.31 (20)	***6.96 ± 0.83 (10)	
	diameter, μm	2.30 ± 0.09 (20)	***0.84 ± 0.10 (10)	
IS	length, μm	7.40 ± 0.16 (20)	***4.20 ± 0.24 (10)	
	diameter, μm	3.06 ± 0.12 (20)	2.94 ± 0.11 (10)	
Myoid	diameter, μm			1.28 ± 0.45 (4)
	length, μm	~0	~0	7.27 ± 1.99 (4)

The data were presented by *mean* ± *standard error* (cells). The *lm*-cones had a shorter and narrower OS and a shorter and rounder IS, compared to regular cones (***p < 0.0001). The *ss*-cones had a much longer myoid.

**Fig 2:**
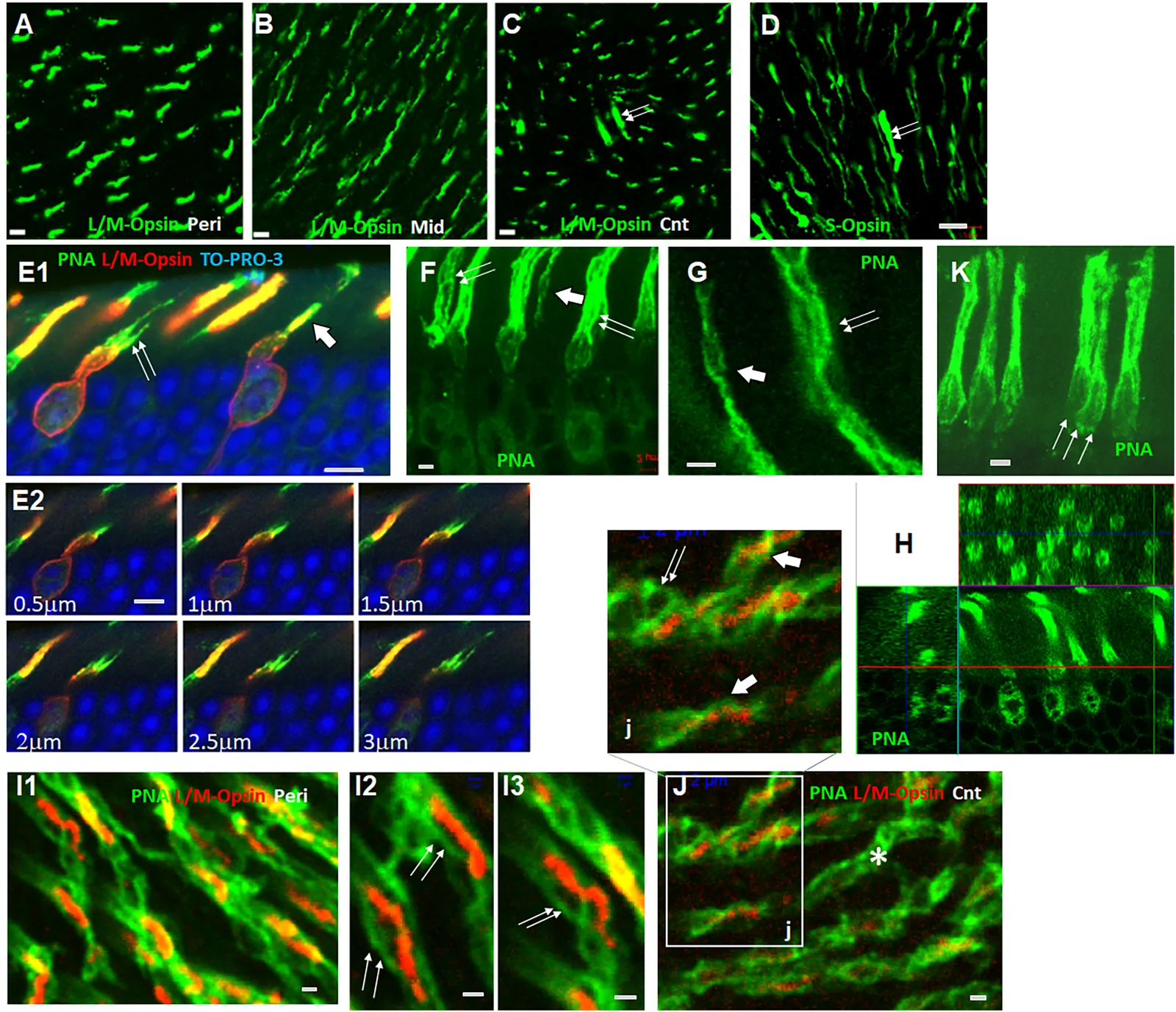
The morphology and immunology of cone OSs and ISs in the mouse retina. **A-D**: Confocal images of flat-mount retinas labeled for L/M-opsin (A-C) and S-opsin (D) show cone OSs with two different calibers. The peripheral ones (A, Peri) are thicker than those in the middle (B, Mid) and central (C, Cnt) retinal regions. **C, D:** Some thicker OSs (double arrow) among many thinner OSs. **E1 and F-H**: Stacked images, each from a series of optical sections. **E**: The retinal slice was triple-labeled for PNA (green), L/M-opsin (red), and TO-PRO-3 (blue). Optical sections (E2) and the stacked image (E1) showed a cone with a thinner single OS (thick arrow) and a cone with a two-unit OS (double arrow). **F, G**: In the retinal slice (F) and flat-mount (G), PNA signals reveal two strings in the thinner OSs (thick arrow) but 3-4 strings in thicker OSs (double arrow). **H**: Orthographic projection of an image stack shows the cross-section of the IS with uneven PNA signals consistent with the strings. **I, J**: The L/M-positive disk region fills the space formed by two PNA strings in the central retina (J, thick arrow) but takes nearly half of the space formed by 3-4 PNA strings (double arrow) in the peripheral (I) and central (J) retina. *: A two-PNA-string-wrapped OS was L/M-opsin-negative. **K**: Two cones attached at the IS and OS. Scale bars are 20 μm in A-D, 5 μm in E, and 2 μm in others. OS: outer segment; IS: inner segment.

TO-PRO-3 labeled nuclei of cones less intensively than rods. Cone somas were oval and much larger than those of rods, and rod somas were nearly round. Each cone contains one soma with 1–4 nuclear regions, often 2–3, which appeared to be separated (Fig 1A2, 1D2 and 1G), and fluorescent signals of PNA (Fig 1D2) and L/M-opsin (Fig 1A2) were present between these regions. Each nuclear region contained one nucleolus intensely positive for TO-PRO-3 (Fig 1D2). The nuclear membrane marker Lamin-B1 [33] revealed one nuclear envelope for most cones (Fig 1G1). Lamin-B1 occasionally revealed two closely attached nuclear compartments (Fig 1G2), and the combined size, appearance, and distance to other cones were comparable to solitary cone nuclei.

PNA stained both L/M-positive cones (Fig 1A–1D) and S-opsin-positive cones (Fig 1H). A small number of cones were entirely positive for S-Opsin, including their OS, IS, soma, and axon terminal, and we termed them as *ss*-cones (Fig 1E and 1F) to distinguish them from the S-cone previously identified by the expression of just S-opsin in the OS. S-cones have not been previously known to include double cones, either. The *ss*-cones showed normal anatomical features at the soma, axon, nuclei, and nucleoli. They took 5.7% of the cones positive to S-opsin, showing a soma located near the third soma row in ONL, a thinner IS, and a rather long IS-soma distance (i.e., myoid [34,35]). Some *ss*-cones also exhibited two shorter OSs positive for S-opsin, revealing novel single and double *ss*-cone subtypes. Table 1 summarizes the IR in cones, and Table 2 compares the morphology of cone subtypes. Our data did not exclude L/M-opsin or S-opsin from locating in *ss*-cones or *lm*-cones, respectively. These cones were present among regular cones (Fig 1E and 1F) without forming clusters, and they were more frequently found in locations of higher cone densities.

Table 2 showed that the OS of *lm*-cones was significantly shorter and thinner than that of regular cones. The IS of *lm*-cones were significantly shorter and rounder compared to regular cones. The *ss*-cones showed a characteristic long myoid between the soma and IS, which was nearly absent in other cones. The characteristic features of *lm*- and *ss*-cones demonstrate novel anatomical cone subtypes. Their higher opsin levels imply relatively higher color sensitivity.

The thickness of OSs varied in regular cones. The disk region identified by L/M-opsin-IR averaged 0.68 ± 0.04 μm and 1.23 ± 0.04 μm in diameter in the intermediate and peripheral retina (Table 3), respectively (p < 0.0001). OSs were 11.26 ± 0.31 μm in length. We further studied the OS structure. The results (Fig 2 and Table 3) identified two OS calibers for the OSs positive for L/M-opsin or S-opsin, which were preferentially distributed in the central and peripheral retina, respectively (Fig 2A–2C, 2I and 2J). For the thin OS, PNA signals revealed two strings twisted together, while for the thick OSs, three or four intertwined strings extending from the OS to the IS (Fig 2F–2J). The texture was unlikely due to sample preparation, because the strings in adjacent OSs showed different twisting patterns. The diameters of the thin and thick OS were around 1–2 μm and 2–3 μm, respectively, and each PNA^+^ string was ~ 0.25–0.35 μm thick. We propose that these strings help to hold OSs in a vertical shape, withstanding the mechanical forces in the ocular environment. Under high magnification, we observed that the two-PNA^+^-string-wrapped OSs were either positive or negative to L/M-opsin (Fig 2J). The L/M-positive OS disk region fully occupied the lumen wrapped in two PNA^+^ strings, but it just eccentrically filled nearly half of the lumen formed by 3–4 PNA strings, seemingly leaving a vertical OS compartment negative for both PNA and L/M-opsin (Fig 2I and 2J). The data suggest that individual thicker OSs may contain an L/M-positive and an L/M-negative compartment.

**Table 3:** Dimensions of cones in the mouse and monkey retina.

	Location	Labeling	OS length μm	OS diameter μm	OS disk diameter μm	
Mouse cones	Intermediate	L/M-opsin	11.26 ± 0.31 (20)	0.68 ± 0.04 (41)		
	Peripheral			*** 1.23 ± 0.04 (24)		
Monkey cones	Central	PNA, L/M -opsin	20.74 ± 0.78 (28)	1.21 ± 0.02 (28)	1.21 ± 0.02 (28)	
	Intermediate (Mid)	PNA		***5.49 ± 0.12 (121)		
		L/M-opsin			***1.58 ± 0.05 (22)	
		Calbindin			***1.67 ± 0.09 (16)	
	Peripheral	PNA		***10.18 ±0.62(18)		
Monkey (Mid) cones OSs, PNA						
	Left PNA^+^ wall, μm	Center span, μm	Right PNA^+^ wall, μm	OS total diameter, μm	Disk zone diameter, μm	Central PNA negative zone, μm

	1.81 ± 0.12 (16)	3.11 ± 0.14 (16)	1.80 ± 0.14 (16)	6.66 ± 0.27 (16)	1.67 ± 0.09 (16)	1.44 ± 0.12 (16)

The data were presented by mean ± standard error (cells). OS: outer segment. ***p < 0.0001 compared to the central retina (monkey) or intermediate retina (mouse)*.*

Cone somas were weakly labeled for L/M-opsin throughout the retina with a nearly even distribution and a density of 13,830, 1,1300, and 8,265/mm^2^ in sampled areas from the central, middle, and peripheral retina (Fig 3), yielding an estimation of 116,883/retina (n = 2) similar to a previous finding in albino mice (117424/retina) [36]. Scanning with the same confocal parameters, we found that cones in the central retina had weaker L/M-opsin-IR in somas but brighter L/M-opsin-IR in nucleoli compared to cones in the middle and peripheral retina (Fig 3). Such general intensity gradient implies an enhanced production of L/M-opsin near the central retina, which may probably be related to the stronger physiological light exposure at the central retina. In addition to this regional gradient, a small number of cone somas (Fig 3 D1, and insets d1 and d5) were labeled more brightly (i.e., *lm*-cones) than adjacent ones (regular cones). The somas of these *lm*-cones were mostly found in the central retina without forming clusters, and their nucleoli were less L/M-opsin-IR (Figs 1 and 3).

**Fig 3:**
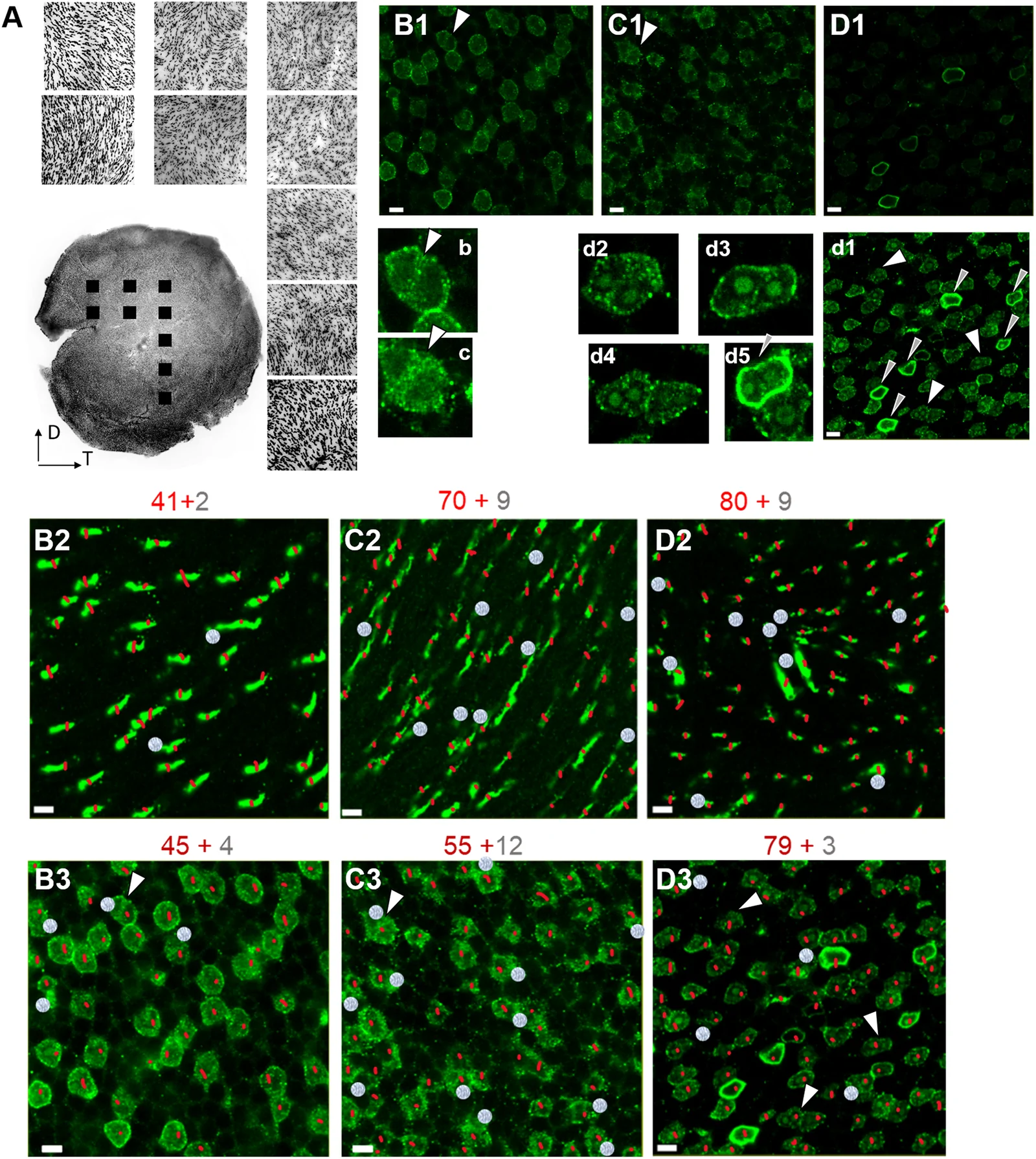
Cone populations positive for L/M-opsin. **A**: A whole retina with nine areas magnified, showing a generally even distribution of L/M-positive cones. D-Dorsal; T-Temporal. **B-D1** were taken at the soma plane from the peripheral (B1), middle (C1), and paracentral retinal (D1) regions, and acquired with the same confocal parameters. Their brightness was enhanced for better visibility and presented in **B-D3,** respectively. Insets: **b** for B1, **c** from C1, and **d1-4** from D1. OSs in these areas are displayed in **B-D2**, respectively, for parallel counting. **D1**: In the central retina, a small number of cone somas (*gray* arrowhead, d1 and d5) are labeled more brightly (*lm*-cones) than adjacent ones (regular cones). Nucleoli are revealed in the central retina (d2-4, D3) but not in middle and peripheral retina (B3, C3, b and c). **B1, C1, B3, and C3**: Regular cone somas are labeled brighter in peripheral and middle retinas. Some somas have a two-chamber-like appearance (b, c, d2, d3 in B-D1; *white* arrowhead in B3 and C3). **B-D2** and **B-D3**: Cells were counted manually by putting dots on them. Red dots and numbers indicate cells identified with high confidence, and gray dots and numbers show OSs or somas identified with uncertainty. **C3 and D3**: Counting the somas of a two-chamber appearance as two cells makes the cell counts close to the confidential OS counts in C2 and D2. **C2, C3, D2, and D3**: Including the very short and thin OSs (gray dots), which were at a variable distance from other OSs, into the OS counts makes the OS counts nearly 10% more than that of somas. **B2 and B3**: OSs are chunkier and counted ~10% fewer than somas in the peripheral retina. Scale bar is 5 μm.

Some L/M-opsin-positive cone somas, as well as the IS and OS compartments (Fig 2K), were closely attached. These somas showed a two-chamber appearance (Fig 3, white arrow and insets b, c, d2, d3), were present throughout the retina, and took nearly 10% of L/M-opsin-positive somas. Counting these somas as two cones makes the counts closer to that of confidently identified OSs positive to L/M-opsin in the same regions, indicating that each cone soma has at least one OS unit. On the other hand, the presumed “paired” cone somas were attached like vertebrate double cones [20] and distributed in the array as single cones (Fig 3C3). Including the very short and thin OSs counted with low confidence made the OSs more than somas (~3.3%) (Fig 3B–3D). The proportion and appearance of these untypical OSs appeared to be consistent with the results on OSs of *lm*-cones stained weakly for L/M-opsin (Figs 1B, 1D and 2E). The data together support the presence of mouse double cones. Different cone types co-existed within individual retinas. We did not find gender-associated differences.

### The dorsal-ventral and temporal-nasal gradients of S-opsin-positive cones in the mouse retina

We studied the distribution and density of S-opsin-positive cones in whole-mount retinas (Figs 4 and 5). In our experimental conditions, retinas dissected under ambient daylight cannot provide reliable signals of L/M-opsin and S-opsin, especially in the central retina, probably due to photo bleaching. For better results, we perform some experiments (2/4 retinas) in dark-adapted conditions under infrared illumination. The average population of cones positive for S-opsin at OSs was 158160 ± 15950 (n = 4), and the ventral-dorsal gradient was two times higher than the temporal-nasal gradient. Based on the data from the parallel counting of somas and OSs of cones (e.g., Fig 3B-D2 and 3B-D3), we corrected the population to 152941 cells/retina, considering that OSs might be counted more than somas (~3.3%).

**Fig 4:**
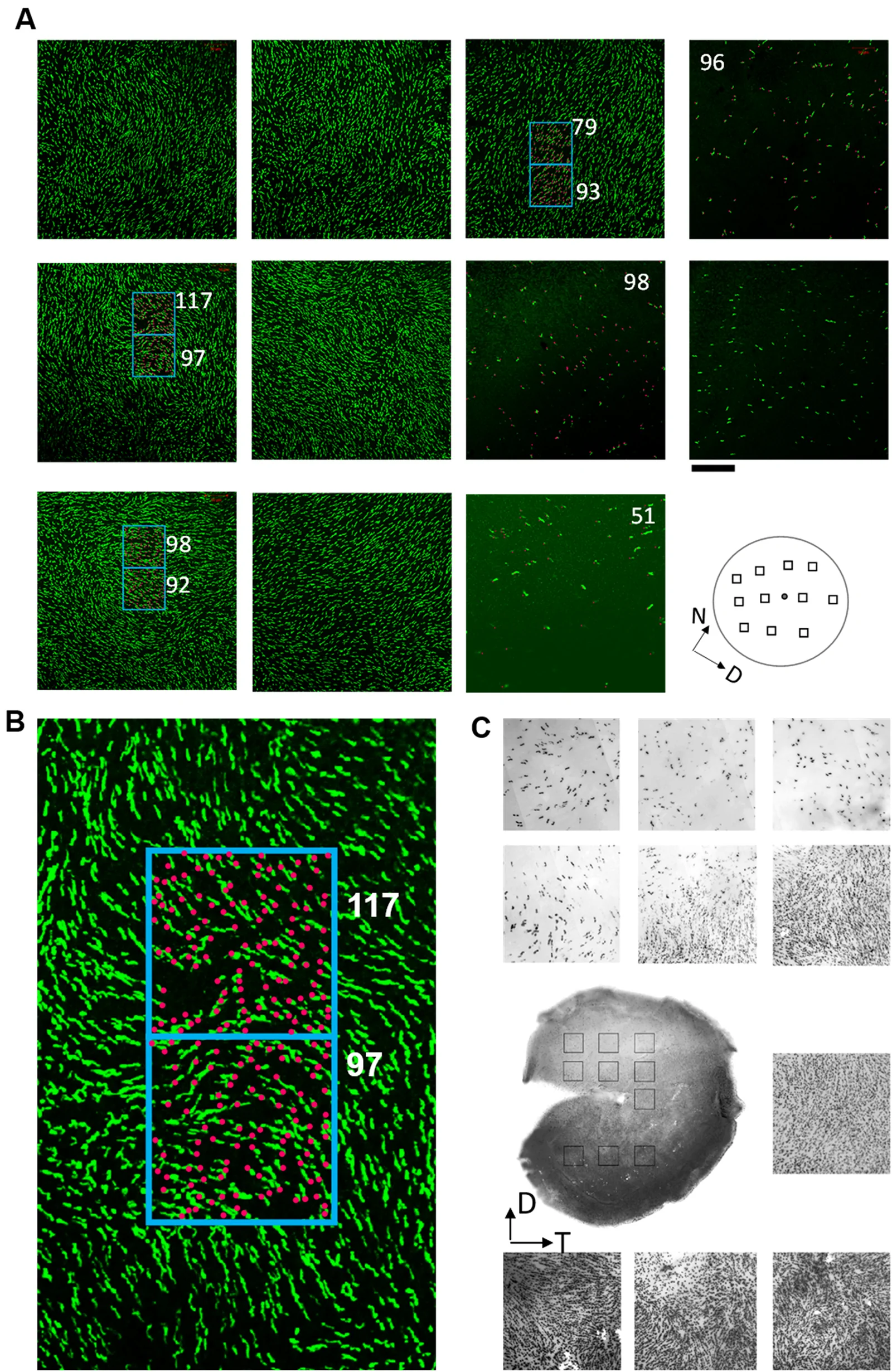
The distribution of S-opsin-positive cones in the dark-adapted mouse retina. Whole retinas were labeled for S-opsin. **A**: A whole retina with 10 areas magnified. **A, B:** Examples of the typical counting approach. The images of a high OS density are counted within smaller areas (blue, 50 x 50 μm) by manually putting red dots on each OS. All OSs in the images were counted eventually. **B**: An area from A under high magnification. Numbers in A and B: OSs counted in blue squares or the entire area. **C**: Another whole retina. The cut was made at the nasal pole. Ventral-dorsal and temporal-nasal gradients are evident. N-Nasal; D-Dorsal; T-Temporal. Scale bar is 50 μm. The data revealed a dorsal-ventral gradient and a temporal-nasal gradient of S-opsin.

**Fig 5:**
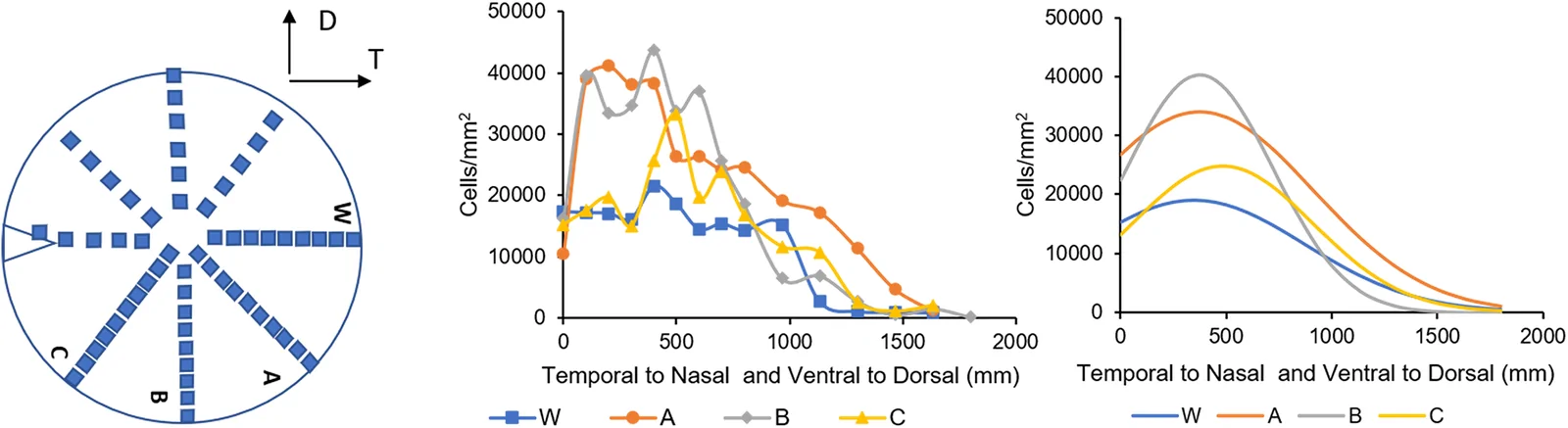
Density of S-cones manually counted from a dark-adapted mouse retina. The triangle at the circle indicates the nasal pole. The OSs were counted on four diameters evenly distributed across the retina, starting from points W, A, B, or C. T: temporal; D: dorsal. The data were well fit to a Gaussian function.

In three retinas, we surveyed the cone density in 5–11 regions between the dorsal and ventral retina (Figs 4 and 5). Individual OSs were identified primarily by the finger shape and a visible intercellular distance. The weaker and shorter OSs were also counted if they were separated from other OSs. In one dark-adapted retina (Fig 5), we sampled cone densities manually from 57 regions along eight evenly distributed straight lines to provide detailed gradient data. The density was the highest in the ventral-temporal retina, 100–700 μm away from the optic nerve. The maximal and minimal cone densities were ~40,000–150 cells/mm^2^ along the ventral-dorsal axis and ~20000–184 cells/mm^2^ along the temporal-nasal axis. Fitting the data (Fig 5) to a Gaussian function yielded the maximum density (*a*), slope factor (*b*), and peak density location (*x*_*0*_) of 40245 cells/mm^2^, 347, and 375 μm (all *p* < 0.0001), respectively, for the ventral-dorsal gradient (B series). They were 19072 cells/mm^2^, 523, and 347 μm (*p*, < 0.0001, = 0.0002, < 0.0001) for the temporal-nasal gradient (W series), 34045 cells/mm^2^, 540, and 374 μm (*p*, < 0.0001, 0.0027, 0.0005) for the A series, and 24848 cells/mm^2^, 427, and 483 μm for the C series (all *p* < 0.0001).

### Untraditional photoreceptors in the adult mouse retina

In tens of mouse retinas (n > 40 retinas) with retinal ganglion cell (RGC) populations retrogradely labeled with Lucifer yellow and Neurobiotin [37,38], we encountered two untraditional photoreceptors (Fig 6). One had a clear OS located in the OS layer (OSL), a soma residing in the outer plexiform layer (OPL) with a large size like RGCs, and a single axon reaching the sublamina b of the inner plexiform layer (IPL) near 70% of the IPL depth. The labeling intensity in its soma, OS, and axon was comparable with that in somas and processes of RGCs. The other atypical photoreceptor showed an OS in the OSL, a soma located in the first soma row of the outer nuclear layer (ONL), and an axon terminal extending beyond the OPL. Its soma and axon were both intensively identified. These untraditional photoreceptors were labeled much more brightly than amacrine cells coupled with RGCs (asterisk in Fig 6B) [37,38]. The data indicate that the cells did not receive Neurobiotin from other RGCs via gap junctions. Hence, we identified them as photoreceptor-RGC hybrids.

**Fig 6:**
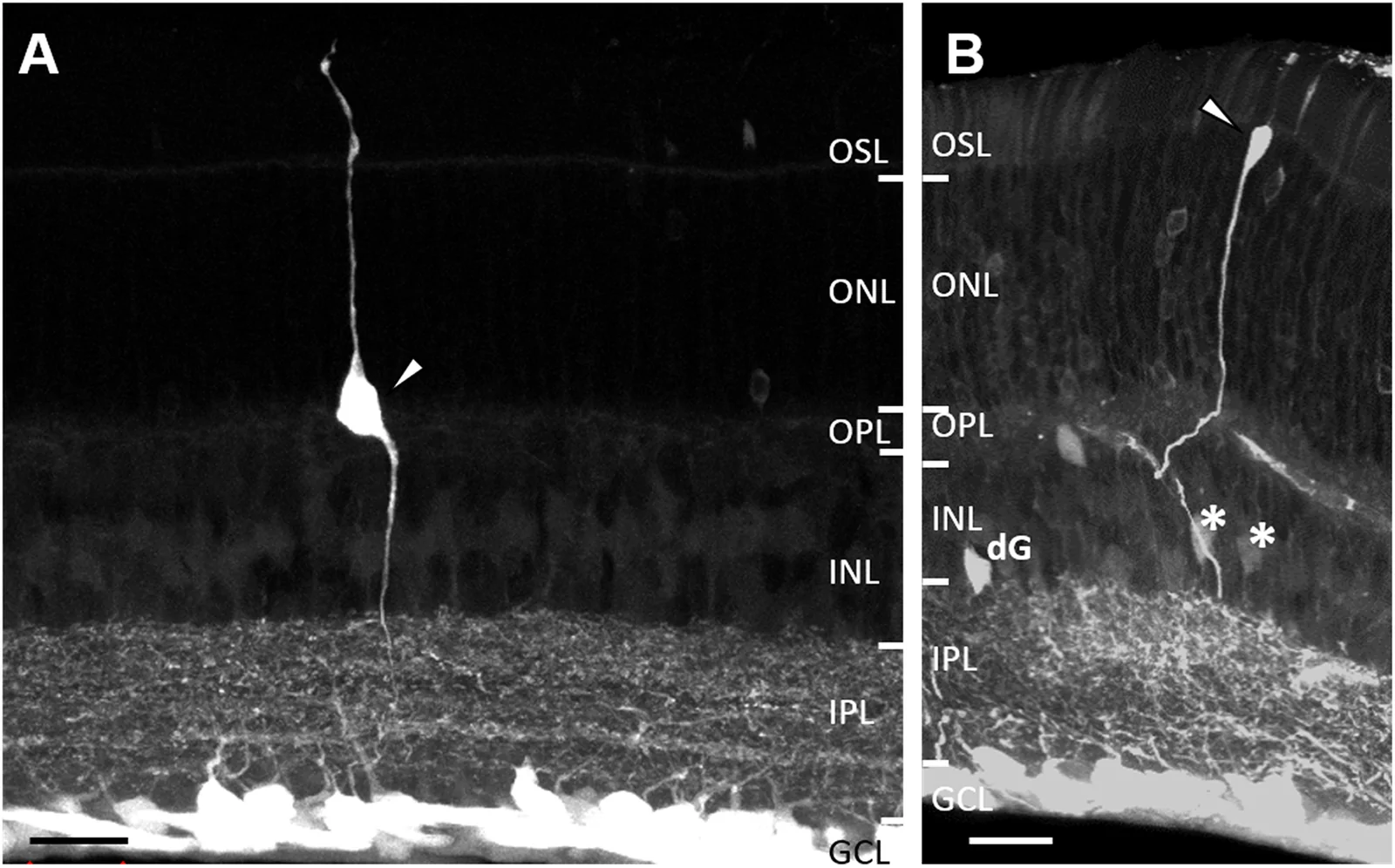
Untraditional photoreceptors in the mouse retina. Retinal slices were retrogradely labeled with Lucifer yellow and Neurobiotin for retinal ganglion cells (RGCs) (white). **A**: A photoreceptor is brightly labeled with an outer segment (OS) residing in the OS layer (OSL) and an axon reaching the inner plexiform layer (IPL). **B**: A photoreceptor is brightly labeled, showing a regular OS, a soma in the first row of the outer nuclear layer, and an axon extending beyond the outer plexiform layer (OPL). The image was overexposed to better visualize the OS. Amacrine cells coupled with RGCs were weakly labeled (asterisks). dG: A displaced RGC. INL: inner nuclear layer; GCL: RGC layer. Scale bars are 20 μm.

### The immvunological subtypes of cone photoreceptors in the primate retina

We studied the morphology of baboon photoreceptors in retinal flat-mounts and slices (n, 5 animals), using immunomarkers and confocal microscopy. Like mouse cones, monkey cone somas were large and longer, containing irregular nuclear regions (Fig 1I). Lamin-B1-IR did not label nuclei of the cones positive for Calbindin D-28K. It identified the nuclear membrane of other photoreceptors and occasionally revealed two nuclear compartments in putative cone somas in the first row of ONL (Fig 1I2).

Calbindin D-28K antibody labeled cone somas brightly and cone OSs moderately (Fig 7A and 7B). 87% of all cone OSs were brightly labeled for L/M-opsin (Fig 7C and 7D). PNA specifically labeled OS sheaths and axonal terminals of all cones, consistent with previous reports from primate [9,39–41] and mouse [10,16] retinas. Moreover, OS disks of most cones (89.5%, n = 114 cells) were brightly positive for PNA, which were even more intensively labeled than cone sheaths (Figs 7A, 7I, 8C and 8D). Disks in 10.5% cone OSs were negative for PNA but positive for calcium-binding protein 5 (CaBP5) (Figs 7J, 7K, 8C and 8D). The data indicate that OS disks express calbindin with PNA in M- and L-cones but CalBP5 without PNA in other cones.

**Fig 7:**
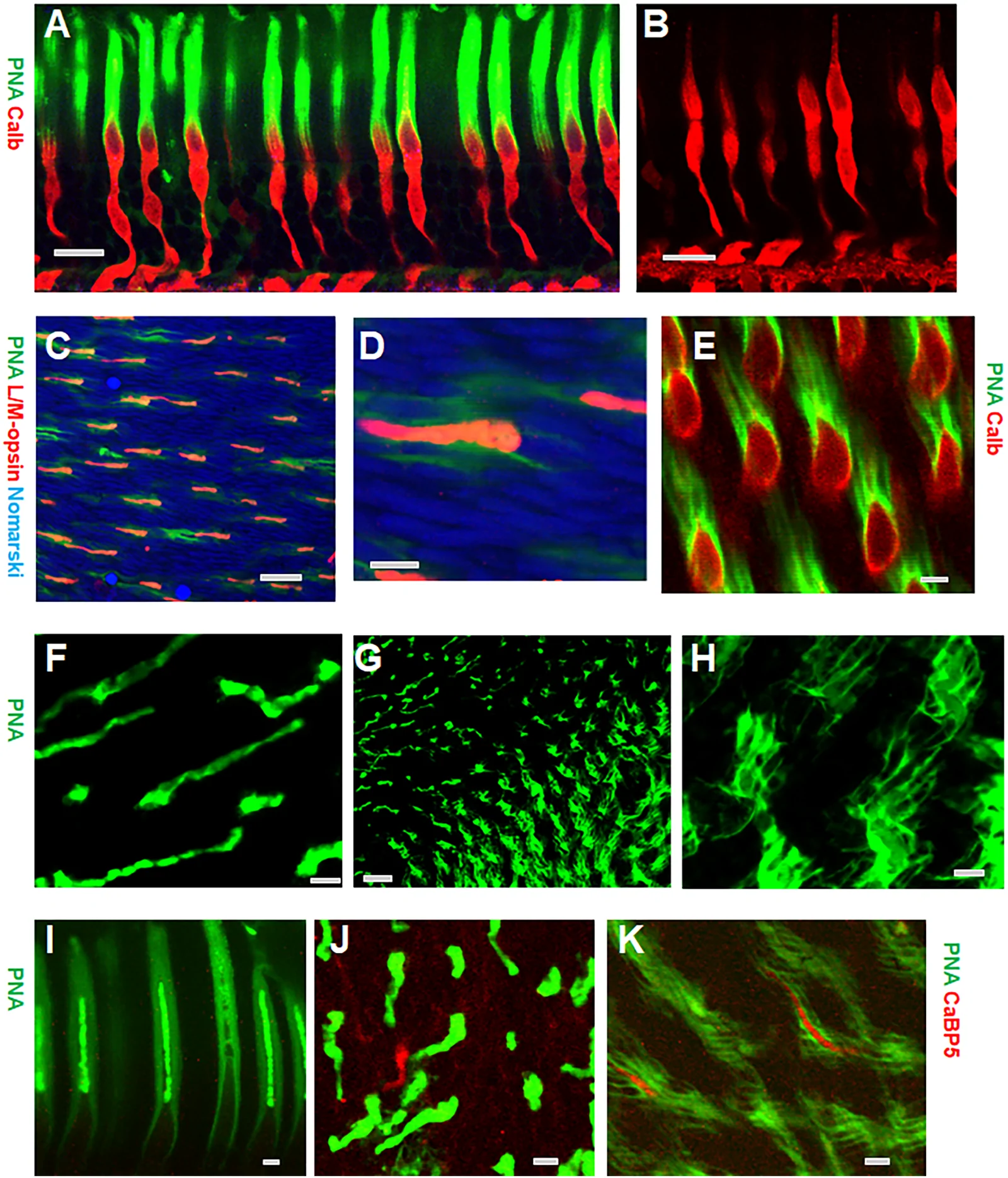
Immunoreactivity (IR) of monkey retinal cones. **A, B, E**: Retinal slices (A and B) and flat-mounts (E) were labeled for PNA (green) and calbindin D-28K (Calb, red). **C, D**: Flat-mount retinas labeled for PNA (green) and L/M-opsin (red). The blue channel shows the Nomarski image. **F-I**: Retinal flat-mounts (F-H) and slice (I) were labeled for PNA. PNA revealed cone outer segments (OSs) are super-thick in the peripheral retina (**H**) compared with the central retina (**F**). **G** shows a clear transitional zone between the cones with chunky and thin OSs. **J, K**: Flat-mount retinas were labeled for PNA and calcium-binding protein 5 (CalBP5, red). **J**: Central retina. **K**: Paracentral retina. The central disk region of most cone OSs are immunoreactive for Calb (A and B), L/M-opsin (C and D), and PNA (I), and some are CaBP5-positive (J and K). PNA-positive grid or wall is revealed in E, G, H, and K. Scale bars are 20 μm in A, B, C, and G and 5 μm in others.

**Fig 8:**
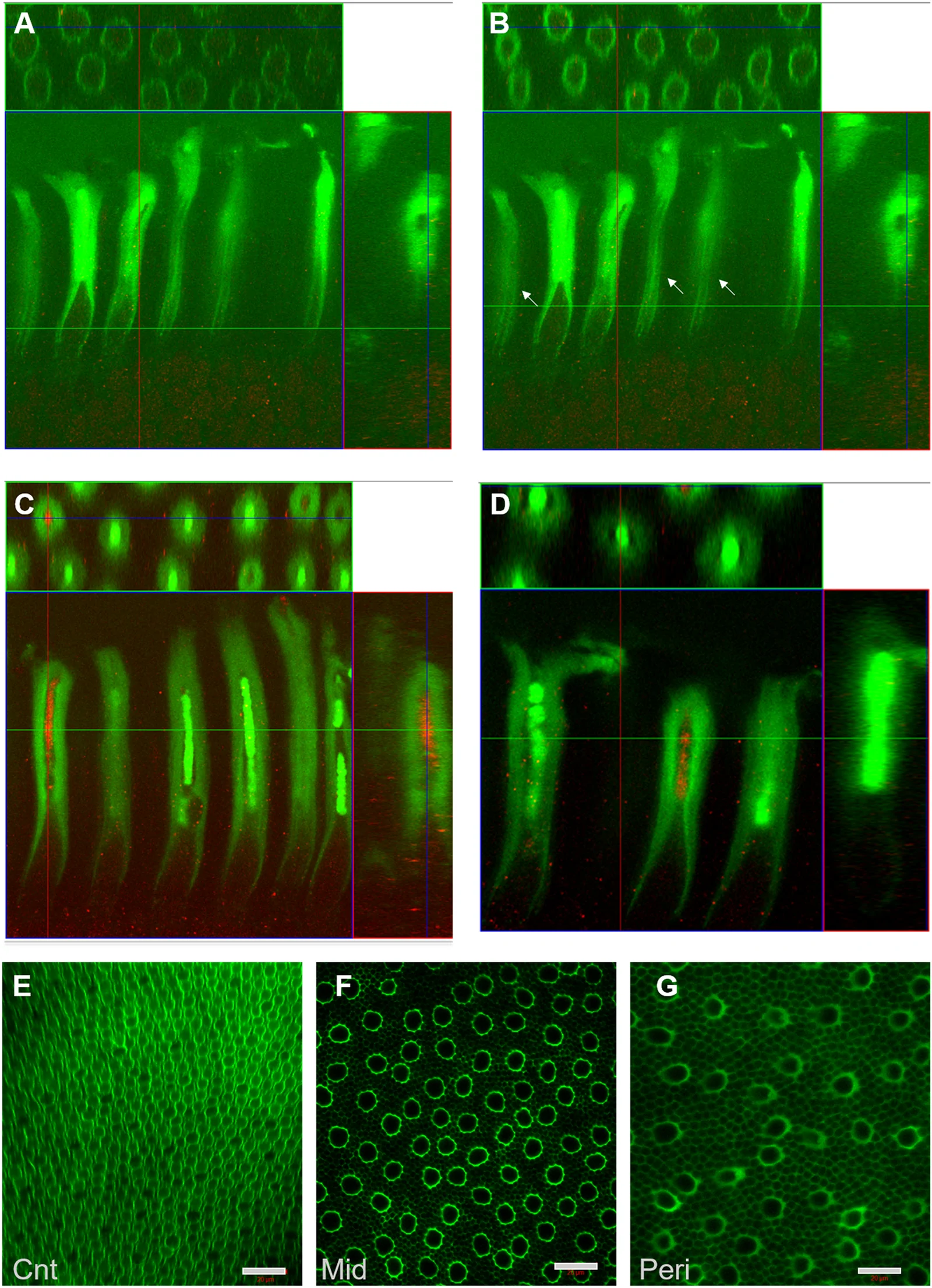
PNA-positive grids in monkey cone inner (IS) and outer segments (OSs). **A-D:** Orthographic projections of stacked confocal images from slices of the intermediate retinal region labeled for PNA (green, A and B) and the calcium-binding protein 5 (CaBP5, red, C and D) at the x-y (central panel), y-z (right panel, red line), and x-z (top panel, green line) planes. Annular PNA signals in the x-z planes are thicker and relatively even at OS levels (C and D) and thinner and uneven at IS levels (A and B). PNA-positive grids are clear in x-y planes on the surface of the OS and IS (A and B). **E-G**: Whole-mount retinas labeled for PNA. The images were taken at the IS plane from the central (Cnt), intermediate (Mid), and peripheral (Peri) regions. Scale bars are 20 μm.

In the paracentral cones of multi-barrel PNA signals, L/M-opsin or CaBP5 IR identified a disk zone as thin (Fig 7K) as that of fovea cones of a single-barrel appearance (Fig 7F and Table 3), and the immunosignals were present only in one barrel of the multi-barrel OSs.

### The structure of baboon cone OSs

We compared the size of cone OSs and ISs in retinal regions (Tables 3 and 4). Cone OSs, including the disk zone and PNA^+^ sheath, as well as the IS diameter and distance, were smaller in the central retina and larger in intermediate regions and the peripheral region, presumably the ventral retina. Under low magnification, PNA-labeled individual cone OSs appeared to be single-barreled (Fig 7F) in the central retina and multi-barreled (Fig 7E, 7G, 7H and 7K) in other regions, and the transitional zone is narrow (Fig 7G). OSs with PNA^+^ sheath were measured 1.21 μm, 5.49 μm (*p* < 0.0001), and 10.18 μm (*p* < 0.0001) in diameter in the central, intermediate, and peripheral retina (Table 3), respectively, and the location-related increase in the OS disk diameter was small, though *p* < 0.0001. In the OS of fovea cones, PNA^+^ walls were not identifiable from OS disk regions, and CaBP5-positive OSs showed no PNA signals (Fig 7J). The data indicate a lack of PNA^+^ sheaths in OSs of fovea cones and a thicker sheath in other retinal regions.

**Table 4:** Cone-rod mosaic at the inner segment plane in the monkey retina.

	Central (Cnt)				Intermediate (Mid)				Peripheral (Peri)			
	Cone	Cone	Rod	Cone	Cone	Cone	Rod	Cone	Cone	Cone	Rod	Cone
	Contacted rod number	D μm	D μm	Nearest distance μm	Contacted rod number	D μm	D μm	Nearest distance, μm	Contacted rod number	D μm	D μm	Nearest distance μm
Mean	0.00	5.24	n.a.	0.00	12.13	8.96	2.74	4.00	10.67	9.87	4.13	7.93
s.e.	0.00	0.16		0.00	0.22	0.19	0.08	0.32	0.16	0.28	0.09	0.76
n	15	15		15	15	15	15	15	15	15	15	15
*p*, Mid vs. Cnt					<0.0001	<0.0001	n.a.	<0.0001				
*p*, Peri vs. Mid									<0.0001	0.013	<0.0001	<0.0001

Retinas were labeled for PNA. n: cells. s.e.: Standard error. n.a.: not applicable. Cnt: Central retina; Mid: Intermediate retina; Peri: Peripheral retina; D: Diameter.

Under high magnification, PNA signals revealed several nearly straight parallel grids of 0.9–0.25 μm-thick along the vertical axis of OSs. The grids extended from the OS to the IS. In the IS, they become thinner and distended outward to provide an enlarged space for the nucleus. The grids divided the OS into vertical zones of nearly equal width, which was particularly clear in the peripheral retina (Fig 7H), providing each cone OS with a multi-barreled impression. In the intermediate and presumable peripheral ventral retina, 4–6 bright PNA^+^ grids were revealed on one side of the OS surface. Based on the dimension of cones, we estimated that each cone likely possessed 8–12 PNA^+^ grids. The diameter of cone ISs became two times larger with progressively longer inter-IS distance from the central to peripheral retina (Figs 7 and 8 and Table 4). Each cone was surrounded by 10–13 rods in the intermediate and peripheral retina, and the thickness of rod ISs was ~ 3–4 μm (Table 4). The number of PNA grids in the cone sheath seemed to be consistent with the number of the surrounding rods.

In the intermediate area, the overall OS diameter revealed by PNA signals averaged 5.49 μm, and the diameter of the OS disk zone identified by PNA or CaBP5 signals was measured 1.6–1.7 μm. In a sampled area (Table 3 bottom, Fig 7I), the overall OS diameter was 6.66 μm, and each PNA^+^ OS wall was 1.8 μm thick, providing a lumen of 3.11μm. This space enclosed the disk zone of 1.67 μm and also a negative zone of 0.7 μm at each side. This negative zone was likely occupied by the OS membrane but appeared to be wider than one layer of plasma membrane, known to be ~ 0.01-μm-thick [42].

## Discussion

### Several novel cone subtypes are observed in the mouse and primate retina

This study identified several novel cone subtypes by immunology and morphology, including double and single *lm*-cones and double and single *ss*-cones in the mouse retina and L/M-opsin/PNA/calbindin-expressing cones and S-opsin/CaBP5-expressing cones in the monkey retina.

Double cones have not been reported in the mouse or monkey retina [21,22]. Cone photoreceptors that look like twins with attached OSs and somas have been identified as double or twin cones (DCs). The DC is the most common cone subtype in fishes, reptiles, and birds [18–20]. Its significance and whether it has unique functions differing from single cones are unclear. It has been suggested that DCs perform achromatic tasks such as luminance, motion, and polarization vision [19], while recent studies have demonstrated that individual members of DCs mediate color vision as independent spectral channels [43]. In this study, we observed cones of two-chamber-like somas, dual OSs positive for opsins, and thick OSs with a twin vertical compartment in the mouse retina. The nuclear membrane marker Lamin-B1 revealed one nuclear package in a great majority of the mouse and monkey cones and two nuclear areas on some occasions. Although cones in the monkey peripheral retina showed a “multi-barrel” OS, we did not find cones with more than one immunologically identified OS. Lamin-B1 did not label calbindin-positive monkey cones in this study, and it was reported to have a curvature-dependent loss in the nuclear envelope [33]. Our data cannot fully exclude the presence of DCs in the monkey retina.

L/M-opsin [9,36] and S-opsin [14,15] signals can reveal some normal cones at the soma, as well as axon terminals useful for identification of cone-bipolar synaptic contacts in literature. Yet, opsin signals in somas and axons were rarely examined in populational studies [2,12], and it has been unclear whether these signals are physiological or related to specific cone subtypes [9,14,15,36]. In this report, we characterized *lm*-cones and *ss*-cones based on their immunoreactivity and morphology for the first time. Their higher opsin levels suggest them as novel cone subtypes likely possessing higher color sensitivity. Most mouse cones are mixed cones, co-expressing M- and S-opsin [12,15], which should take 97–90% of all cones, given that pure S-cones take 3–10% of total cones [14,15,44,45]. Controversially, other researchers reported 40–84% for mixed cones and 10–26% for pure S-cones [2,15]. In our results, *lm*-cones took 7.6% of L/M-opsin-expressing cones, and *ss*-cones took 5.7% of S-opsin-expressing cones. The populations differ from that of previously identified cone subtypes, and we identified them as novel subtypes. It is uncertain whether *lm*-cones are counterparts of primate L-cones.

Although opsins are usually found in OSs, in this study, most mouse cones showed weak L/M-opsin-IR at the soma (Fig 3) and intense IR at the OS, indicating that opsin signal in the soma is not a definite sign of damage or pathology. Researchers have reported similar phenomena in multiple mammalian species, including the mouse [9,14,15,36], which were presumably physiological [46–51]. In pathological studies, local degenerating cones may show intense opsin signals accompanied by axonal pathology [52]. However, our *lm-* and *ss*-cones had a unique and healthy appearance and were present among neighboring cones of normal morphology, arguing against damage. Living retinas may be physically irregularly damaged or evenly photo-bleached during dissection, which, on the other hand, has little chance of selectively affecting individual cone somas or OSs without damaging their neighbors at the same focal plane. Moreover, we used retinal slices 45 μm thick and often scanned individual cones with a series of confocal optical sections for completeness, without taking images from the very surface. We also observed in patch-clamp studies that acute damage to living retinal neurons usually manifested as varicosities in axons and dendrites and a rounded, expanded soma. The *lm*- and *ss*-cones in our results did not show these types of injuries.

It has been poorly understood how opsin expression is regulated under physiological conditions. A few studies indicate that thyroid hormone [53] and orange light [54] may enhance the expression of M- or L -opsin but suppress that of S-opsin [53], and cones in nocturnal [48] and developing animals [46,47] appear to show more opsin signals in the IS, which identify three physiological regulation mechanisms, 1) endocrinologic, 2) environmental/adaptive, and 3) developmental. In mice lacking rhodopsin and RPE65, L/M-opsin observed in the somas of peripheral cones was thought to be “mislocalized” and corrected by peritoneal injection of 11-cis-retinal [55]. RPE65 is a protein highly expressed in the RPE and involved in the generation of 11-cis-retinal, and exogenous 11-cis-retinal can be utilized by cones [56]. These results are consistent with the notion that cone somas can adaptively upregulate opsin synthesis when the physiological need for photopigments increases. The nucleolus is a prominent domain in eukaryotic cells, lacking a delimiting membrane. It is primarily responsible for the biogenesis of ribosomes [57,58], where the translation of mRNA information into proteins [59] takes place. This study showed brighter L/M-opsin signals in the nucleolus than in the cytosol in the central cones, but opposite results in cones in the peripheral and middle retinas, aligning with an increased need and active production of opsins in central cones. Given that the protein moiety of photopigments is synthesized primarily in the soma [57–61], we think that the brighter opsin signal in somas and axons of *lm*- and *ss*-cones is not pathological and likely mediates relatively higher color sensitivity in these cones.

Retinal ganglion cells rely on at least two levels of synapses to receive visual signals, one of which is in the OPL and the other is in the IPL [62,63]. Here, we revealed two photoreceptor-RGC hybrids in the mouse retina by retrograde labeling of RGCs. These hybrids possess an OS as photoreceptors, a large nucleus in the OPL or ONL, and an axon extending beyond the OPL, in one case to the IPL. The cells may represent a development error, though.

In the monkey retina, our data first identified strong PNA signals in the disk zone of 89.5% of cone OSs, and 10.5% of OSs expressed CaBP5 but not PNA. L/M-opsin-positive disc zones express PNA, indicating that M- and L-cones express PNA in OS disks, and S-cones express CaBP5 in OS disks. Further studies will be required to define the physiological significance of PNA and CaBP5 in cone subtypes. Moreover, we observed that 87% of cone OSs expressed L/M-opsin, resembling the population of mouse cones that possess L/M-opsin (~90%) [14,15,44,45]. It indicates that primate and mouse retinas weigh the longer wavelengths more than blue light.

### S-opsin shows a Gaussian distribution with the dorsal-ventral gradient two times higher than the temporal-nasal gradient, and the rod-cone coupling may be affected by the cone density and the rod-cone mosaic

S-opsin in the mouse retina has been known for a ventral-dorsal (V-D) gradient [12–15]. The related reports have disagreements and lack a detailed quantification for the V-D and temporal-nasal (T-N) gradients of S-opsin [2,15]. Ortin-Martinez and colleagues [2] reported differential S-opsin distribution in three retinal territories and revealed the V-D and T-N gradients in both pigmented and albino mice. On the other hand, Nadal-Nicolas and colleagues [15] examined horizontal gradients of S-opsin at two vertical locations (V, D) without reporting a clear T-N gradient in albino mice or details on the V-D gradient in both pigmented and albino strains. Our results quantitatively revealed the V-D and T-N gradients of S-opsin, which showed a Gaussian distribution. The density peak was present at the ventral-temporal retina 100–700 μm away from the optic nerve, and the V-D gradient was two times higher than the T-N gradient. Since OSs, especially longer ones, may be twisted and counted more than once, we performed parallel counting of somas and OSs in some retinal regions. Based on the results, we corrected the number of S-opsin-positive cones to 152,941 per retina. This result is generally consistent with the previous reports [12,14,15,36] (e.g., 146,682/retina in albino mice [2]) but is higher than the count in pigmented mice (119,616/retina) [2]. Our maximum cone density of ~40,000/mm^2^ is equal to that of rats [36] but higher than earlier observations in mice [1,2]. The higher estimation may be attributed to our dark-adaptation-enhanced labeling of opsins, mounting methods, and manual counting. We mounted whole retinas with pieces of 180-μm-thick filter paper [37] to minimize the artificial flattening-associated surface expansion.

Meanwhile, the findings on M/S, M-, and S-cone populations in the literature have discrepancies. Nearly all murine cones are mixed M/S-cones (97–90%), which co-express S- and M-opsin, except in the far dorsal retinal region [12,14], and the pure S-cones was estimated to be ~ 3–5% [14] in the dorsal retina or 7–8% [36] and less than 10% of all cones [15]. Controversially, later data showed a fraction of 40–84% for mixed cones, 10–26% for pure S-cones, and 34–37% or 1–3% (albino mice) for pure M-cones [2,15]. Our estimated population of M-opsin-positive cones was consistent with that in albino mice [2], supporting a higher fraction of pure S-cones. The automatic [2,15] and manual counting approaches differ in the sensitivity of detecting low levels of opsins. Plus, animals in the reports lived under variable stress and light levels. Stress may reduce thyroid hormone [64] to activate the expression of S-opsin but represses M-opsin expression [53], and orange light also increases L-opsins [54]. These factors likely contribute to the inconsistency in reported cone populations.

In the primate retina, we observed a dramatic shift in the cone thickness between the central retina and adjacent areas. Rods are absent in the fovea of the monkey retina, and cones near the central retina have fewer rods surrounding them because of their small diameters and a lower rod density. However, in the peripheral retina, we observed 11–12 rods surrounding individual cones. The rod-cone coupling has been widely accepted as the secondary rod pathway [65,66] and presumably serves to enhance the light sensitivity of cones. It is to be investigated how the density and the arrangement of the rod-cone mosaic affect the secondary light pathway in the central and peripheral retina.

### The PNA-positive sheath is more likely the wall of cones and much thicker in the peripheral retina

Our results demonstrate a thicker PNA^+^ sheath in peripheral cone OSs and a thinner PNA^+^ sheath in central cone OSs in the mouse and monkey retinas (Fig 9). The OS of cones has been described by a rather narrow cone with a set of comb-like discs [22,67], which appear to be quite vulnerable to physical stress. PNA has been widely used to label cones [9,10,16,41], and the PNA^+^ component of cones has long been identified as a part of the IPM instead of a cellular component of cones. In this study, the OS disk zone and the sheath were both positive for PNA in most primate cones. The sheath covered the OS and IS in mouse and monkey cones with a string- or grid-like structure. The strings in the mouse cones look flexible and are twisted and intertwined. The grids in the monkey cones smoothly continue from the OS to IS with similar distance, IR, and thickness. The uniformity of PNA^+^ strings or grids indicates that the PNA^+^ sheath is more likely to serve as a wall of cones instead of the IPM. This structure of cones could presumably keep cones straight and well-oriented.

**Fig 9:**
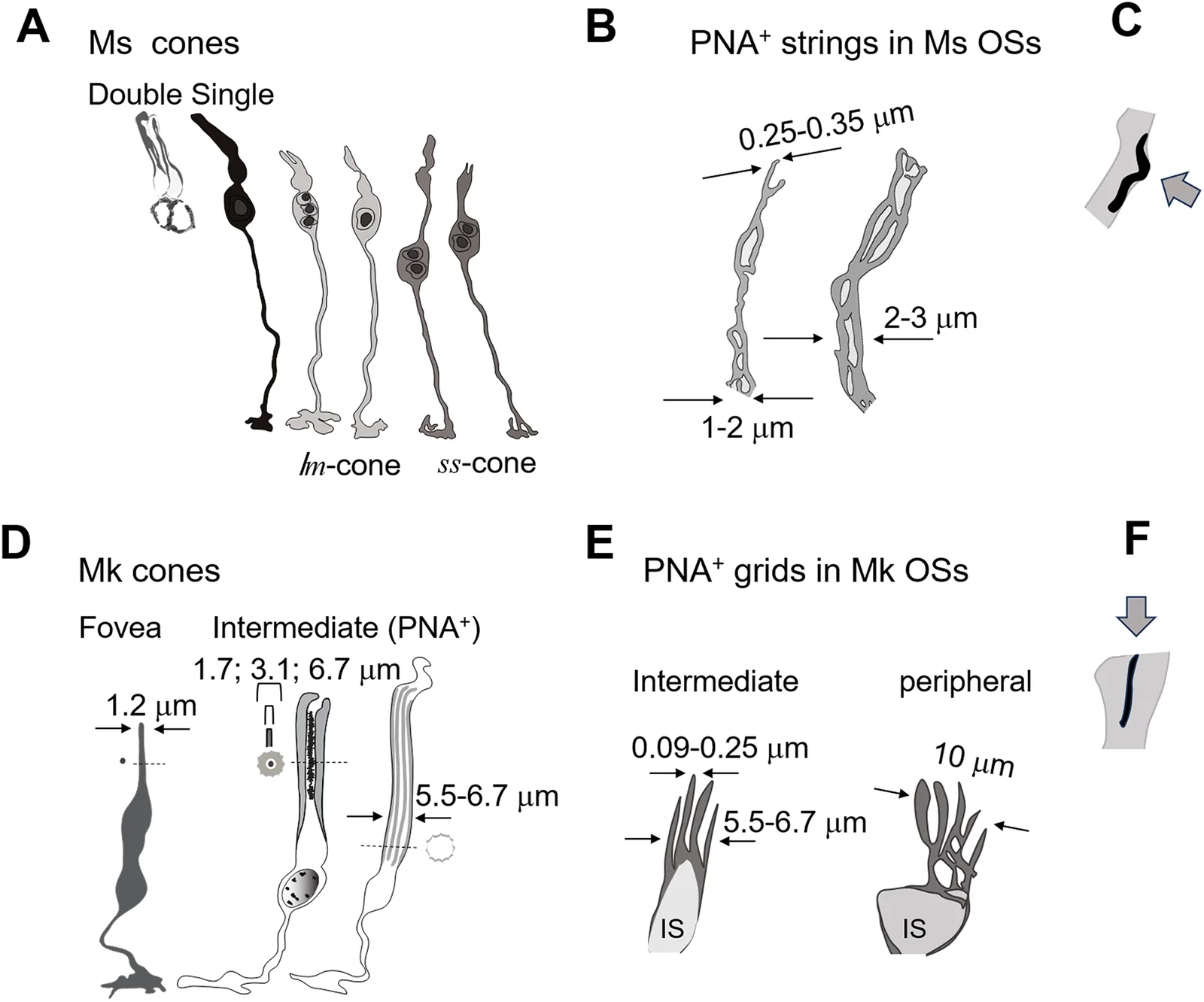
Diagrammatic summary. **A**: Mouse (Ms) cones contain single (s) and double (d) cones with either attached somas or dual OSs. **B**: Ms OSs had two calibers, containing 2 or 3-4 PNA^+^ strings (dark gray). **D-F**: Monkey (Mk) cones. **D**: PNA^+^ grids (light gray) were nearly absent in OSs in the fovea. **E**: OSs were thick in the peripheral retina and contained more PNA grids (dark gray) (in flat-mounted retina). **C, F**: The L/M-positive OS disk region (black, bold arrow) eccentrically filled half of the thick PNA^+^ OSs (gray) in mouse retinas (C), and the disk region was much thinner relative to the PNA^+^ OS (gray) in monkey retinas (F).

Some data have indicated that the PNA^+^ sheath plays a role in macular degeneration [27]. With an absence or thinner PNA^+^ sheath, cones in the primate and mouse central retina are not well supported against physical stress compared to cones in other locations. How this factor contributes to macular degeneration is worthy of further study.

We measured cone structures with confocal microscopes. Confocal microscopy enables us to study larger retinal areas and find less common cell types, but it provides only sub-micrometer resolution. We processed and mounted our samples in aqueous environments, which prevented tissue shrinkage associated with dehydration procedures commonly used in electron microscopy (EM) and many histological labeling methods. Meanwhile, we examined fluorescent signals, which can inflate the apparent size of structures because the signals glow and bleed light into nearby regions. Thus, our measurements of subcellular cone structures are likely slightly larger than their actual sizes and those measured in dehydrated tissues with EM or bright-field microscopes. Mice are considered to mature at 3 months of age [68]. We tested mice at and over this age, but our results cannot completely rule out development-associated variation in cones.

## Methods and materials

### Animals

The mice used in this study were C57BL/6J mice, 3–8-month-old males and females, purchased from Jackson Laboratory (Bar Harbor, ME). All experimental procedures used in this study followed the NIH animal care guidelines and ARVO Statement for the Use of Animals in Ophthalmic and Vision Research and were approved by the Baylor College of Medicine Animal Care and Use Committee. Mice were anesthetized with an intraperitoneal injection of ketamine (200 mg/kg) and xylazine (10 mg/kg). Some mice (9 mice) for studying cones were dark-adapted, and others (15 mice) were light-adapted. Most retinas used for retrograde labeling of retinal ganglion cells (RGCs) were dark-adapted (> 40 mice). The eyes were enucleated after the animals were deeply anesthetized and decapitated. Retinas were immediately dissected in oxygenated Ames medium at room temperature [9,69]. The dark-adapted eyes were dissected under infrared illumination and fixed before further processing. Isolated monkey retinas used in this study were from qualified and licensed third-party sources [9]. Baboon (*Papio cynocephalus anubis*) eyes (five animals between 7–14 years) were enucleated within 10 minutes after the animal had been overdosed with sodium pentobarbital (50–100 mg/kg, IV) by other investigators at the conclusion of experiments that did not involve the eyes. The eyeballs were hemisected, and eyecups with attached retinas were incubated at room temperature in oxygenated Ames medium for 3–5 hours before retinal dissection [70]. Monkey retinas were cut into 3 x 3 mm (9 mm^2^) pieces and then sectioned with a vibratome. Data on the baboon retina were obtained from the paracentral, intermediate, and peripheral regions (~3 mm, 4–7 mm, and >7 mm away from the fovea, respectively).

All retinas were fixed in 4% paraformaldehyde in phosphate buffer (pH 7.4) for 1–2 hours at 4˚C. Other procedures followed previously established protocol [9,69].

### Primary antibodies

Antibodies against calbindin D-28k included two rabbit polyclonal antibodies [21,71] from Swant (Switzerland, CB38, 1: 1000) and Sigma (rabbit, AB1778, 1: 500) and a mouse monoclonal antibody (Sigma, C9848, 1: 100) [9]. Calbindin D-28k is named so due to its molecular weight. The IR to the Swant antibodies was absent in the brain of calbindin D-28k knockout mice [71]. The immunoblot of brain homogenates from the mouse, rat, and monkey with the monoclonal antibody revealed a single band at 28 kDa in MDBK cell extract. The antibody did not react with other members of the EF-hand family, such as calbindin D-9K, calretinin, myosin light chain, parvalbumin, S-100a, S-100b, S-100A2 (S100L), and S-100A6 (calcyclin). The purified polyclonal rabbit anti-S-opsin and rabbit anti-L/M (R/G)-opsin antibodies (AB5407 and AB5405, respectively, 1: 200) were purchased from Millipore-Sigma (Burlington, MA). They were raised using recombinant human S-opsin and L/M-opsin, respectively. These two antibodies primarily and intensively label the OS of cones in the mouse and monkey retinas as previously reported [12,14,15] (see results), supporting the specificity. The anti-calcium-binding protein 5 (rabbit, CaBP5, 1: 500) was a generous gift from Dr. K. Palczewski in the Department of Ophthalmology, University of Washington [73]. Lamin-B1 [33] polyclonal antibody was purchased from Thermo Fisher Scientific Inc. (Sugarland, TX) (PA595850, 1: 500). It was raised from rabbits with a synthetic peptide containing aa1–100 of human Lamin-B1. The antibody identified a single band at ~70 kDa from mouse brain lysates. Fluorescein-labeled PNA (1: 50, Vector Laboratories, Burlingame, CA) was used in the primary antibody diluents. It specifically labeled cones as previously reported labeled [9,10,16,41].

The staining patterns of antibodies and dyes in our results were similar to those in the previous reports [9,10,12–16,21,41,72]. Controls were also processed with blocking peptides or without primary antibodies. All negative controls showed no positive results.

### Immunocytochemistry

Retinal tissues were fixed with 4% paraformaldehyde in phosphate buffer (pH 7.4) for 1–2 hours at 4˚C. They were then blocked with 10% donkey serum (Jackson ImmunoResearch, West Grove, PA) in TBS (D-PBS with 0.5% Triton X-100 (Sigma-Aldrich) and 0.1% NaN3 (Sigma-Aldrich)) for 2 hours at room temperature or at 4˚C overnight to reduce nonspecific labeling. Whole mouse retinas and small pieces of monkey retinas were embedded in low-gel-point agarose (Sigma-Aldrich) and trimmed into a 10 x 10 x 10 mm^3^ block. The block was glued onto a specimen chamber mounted on a vibratome (Pelco 102, 1000 Plus; Ted Pella, Inc., Redding, CA) and subsequently cut into 40-μm-thick vertical sections in PBS solution [37]. For staining, retinal tissues were incubated in primary antibodies in the presence of 3% donkey serum-TBS for 3–5 days at 4˚C. After several rinses, they were transferred into Cy3-, Cy5-, or Alexa Fluor 488-conjugated streptavidin (1:200, Jackson ImmunoResearch), with Cy3- and/or Cy5-conjugated secondary antibodies (1:200, Jackson ImmunoResearch) and/or Alexa Fluor 488-conjugated secondary antibodies (1:200, Molecular Probes, Eugene, OR), in 3% normal donkey serum-TBS solution at 4˚C overnight. A nuclear dye, TO-PRO-3 (0.5 μL/mL, Molecular Probes, Eugene, Oregon), was used with the secondary antibody to visualize nuclei in retinas. After extensive rinsing, retinal preparations were cover-slipped. Two small pieces of filter paper (180-μm thick, MF-membrane filters; Millipore, Billerica, MA) were mounted beside flat-mount retinas to prevent them from being over-flattened.

### Confocal microscopy

This study used Zeiss confocal microscopes (LSM 510 and LSM 800, Carl Zeiss, Germany) and imaging software for morphological observation. Samples were examined under 40x and 60x oil lenses with a vertical resolution of 0.4–1.2 μm at regular line-scan and frame-scan modes. A series of optical sections was scanned over targeted cells, including the soma and all processes, for better visualization of the morphology. The previously established techniques were used for retrograde-labeling of the retinal ganglion cell population [37,38] and the survey of cone density in flat-mounted retinas [37,38]. For presentation purposes, confocal micrographs were further processed in Photoshop software (Adobe Systems Incorporated, San Jose, CA), typically by enhancing contrast and selecting color channels, to provide better visibility to light-adapted human eyes.

### Statistical analysis

Data were analyzed by Sigmaplot software (v12, Systat, Point Richmond, CA), Zeiss confocal software (Zeiss), Photoshop (Adobe), and Microsoft Excel and presented as *mean ± s.e*. (n). The two-tailed Student *t-test* was used for analyzing statistical significance between paired data groups. The density of S-opsin positive cones was fitted to a Gaussian function


$$\begin{array}{c} f(x)=a{exp}^{{\left[-\text{0}.\text{5}(\frac{x-x_{\text{0}}}{b}\right)}^{\text{2}}]} \end{array}$$

for quantification of the dorsal-ventral and temporal-nasal gradients. The α level to reject the null hypothesis was 0.05.
